# Performance of a highly sensitive rapid diagnostic test (HS-RDT) for detecting malaria in peripheral and placental blood samples from pregnant women in Colombia

**DOI:** 10.1371/journal.pone.0201769

**Published:** 2018-08-02

**Authors:** Ana María Vásquez, Ana Catalina Medina, Alberto Tobón-Castaño, Maritza Posada, Gabriel Jaime Vélez, Ana Campillo, Iveth J. González, Xavier Ding

**Affiliations:** 1 Grupo Malaria, Facultad de Medicina, Universidad de Antioquia, Medellín, Colombia; 2 Foundation for Innovative New Diagnostics (FIND), Geneva, Switzerland; Université Pierre et Marie Curie, FRANCE

## Abstract

**Background:**

Pregnancy poses specific challenges for the diagnosis of *Plasmodium falciparum* infection due to parasite sequestration in the placenta, which translates in low circulation levels in peripheral blood. The aim of this study is to assess the performance of a new highly sensitive rapid diagnostic test (HS-RDT) for the detection of malaria in peripheral and placental blood samples from pregnant women in Colombia.

**Methods:**

This is a retrospective study using 737 peripheral and placental specimens collected from pregnant women in Colombian malaria-endemic regions. Light microscopy (LM), conventional rapid diagnostic tests (Pf/Pv RDT and Pf RDT), and HS-RDT testing were performed. Diagnostic accuracy endpoints of LM, HS-RDT and RDTs were compared with nested polymerase chain reaction (nPCR) as the reference test.

**Results:**

In comparison with nPCR, the sensitivity of HS-RDT, Pf RDT, Pf/Pv RDT and LM to detect infection in peripheral samples was 85.7% (95% CI = 70.6–93.7), 82.8% (95% CI = 67.3–91.9), 77.1% (95% CI = 61.0–87.9) and 77.1% (95% CI = 61.0–87.9) respectively. The sensitivity to detect malaria in asymptomatic women, was higher with HS-RDT, where LM and Pf/Pv RDT missed half of infections detected by nPCR, but differences were not significant. Overall, specificity was similar for all tests (>99.0%). In placental blood, the prevalence of infection by *P*. *falciparum* by nPCR was 2.8% (8/286), by HS-RDT was 1% and by conventional RDTs (Pf RDT and Pf/Pv RDT) and LM was 0.7%. The HS-RDT detected placental infections in peripheral blood that were negative by LM and Pf/Pv RDT, however the number of positive placentas was low.

**Conclusions:**

The sensitivity of HS-RDT to detect *P*. *falciparum* infections in peripheral and placental samples from pregnant women was slightly better compared to routinely used tests during ANC visits and at delivery. Although further studies are needed to guide recommendations on the use of the HS-RDT for malaria case management in pregnancy, this study shows the potential value of this test to diagnose malaria in pregnancy in low-transmission settings.

## Introduction

Malaria in pregnancy (MiP) is associated with several adverse outcomes in mother, fetus, and newborn, including maternal anemia, miscarriage, premature delivery and low birth weight [1]. In the Americas region, a total of 562,800 malaria cases were reported in 2016, 15.3% of them in Colombia [2]. It is estimated that at least half million women at reproductive age live in two of the most endemic areas of this South American country (Nariño and Chocó) [3], being at risk of malaria infection.

In Colombia, MiP can be caused by both *Plasmodium vivax* and *Plasmodium falciparum* [4–7], the latter being responsible for the most severe consequences [6,8,9]. Early detection and effective management of infections is crucial for reducing the burden of MiP and for preventing malaria-related pregnancy complications. This is particularly relevant in low-malaria transmission areas such as Colombia, where intermittent preventive treatment is not recommended [10].

Light microscopy (LM) remains the standard of practice for the diagnosis of suspected malaria prior to treatment [2]. Nevertheless, LM presents several limitations, including a reduced sensitivity for detecting low-density infections [11]. It is increasingly appreciated that, in low-transmission settings, a great proportion of asymptomatic cases are caused by low-density blood-stage malaria infections [12,13]. In addition, pregnant women frequently present low-density parasitaemia due to sequestration of *P*. *falciparum* in the placenta and their subsequent circulation at low-density levels in peripheral blood. Furthermore, the absence of clinical signs decreases the probability of diagnosing malaria cases which, together with these low- and fluctuating parasite densities, makes diagnosis by LM challenging [14,15].

Alternatives to LM include the detection of parasite antigens in peripheral blood by rapid diagnostic tests (RDTs) [16]. RDTs are lateral flow immunochromatographic dipstick assays that detect either one or more *Plasmodium* proteins. Antigens detected by commercially available RDTs include histidine-rich protein 2 (HRP2), aldolase and parasite lactate dehydrogenase (pLDH) [16–18]. The use of RDT have facilitated access to malaria diagnosis outside health facilities in peripheral communities, as less training and investment in infrastructure is required when compared with LM and molecular techniques [19].

While RDTs have been extensively evaluated for the diagnosis of malaria among non-pregnant population, few data exist on the accuracy of this test for screening MiP, especially in low-transmission settings outside sub-Saharan Africa [19–22]. In these endemic areas, several studies have shown that RDTs display a sensitivity similar to LM, having both an improved performance when detecting malaria in symptomatic pregnant women as compared to asymptomatic ones [21,22]. Nonetheless both, LM and RDT, fail to detect a large proportion of low-density infections identified by polymerase chain reaction (PCR), mainly in asymptomatic pregnant women screened during antenatal care (ANC) visits [20,21].

Recently, a new HRP2 specific highly sensitive RDT (HS-RDT) has been made available (Alere™ Malaria Ag Pf ultra-sensitive RDT). Using the same immunocrhomatographic cassette platform and volume requirements, the HS-RDT displays about ten-fold lower limit of detection (LoD, ~80 pg/mL) than current best-in-class RDTs [18,23]. When using blood specimens from asymptomatic individuals, recent studies showed an improved sensitivity of the HS-RDT compared with conventional RDT in Myanmar (44% vs. 0%, respectively) and Uganda (84% vs. 62%), suggesting that the new test could be a useful tool for malaria elimination strategies [24].

Considering the improvement in LoD, the easiness to use, and the fact that the HS-RDT fulfills many of the ASSURED criteria [25] and end-user requirements [26,27], the new test could be an optimal tool for detecting malaria in pregnant women naturally presenting low-density infections. The purpose of this study is to evaluate the performance of the new HS-RDT for the detection of *P*. *falciparum* in peripheral and placental blood specimens collected from pregnant women in Colombia.

## Materials and methods

### Study design and population

A retrospective descriptive study was conducted to evaluate the performance of the HS-RDT for screening malaria in peripheral and placental blood samples from pregnant women, compared with LM and conventional RDTs, and using nPCR as the reference standard. The current study used data and samples collected in the frame of a larger cross-sectional project conducted from May 2016 to September 2017 in Colombia, aimed to characterize asymptomatic infections in Colombian pregnant women and to assess the impact of these infections in malaria-related adverse pregnancy outcomes. A total of 766 pregnant women self-presenting at local hospitals for ANC visit, at delivery or seeking medical care for suspected malaria were consecutively recruited. Women, aged ≥ 15 years old, at any gestational age, and living in peri-urban municipality areas or rural areas with malaria transmission were considered eligible for the study. Each participant was enrolled only once in one of the two study groups (ANC or delivery). Overall 737 women, who provided enough samples to perform all laboratory testing, were included in the current study.

### Ethics statements

All samples were obtained in the frame of a cross-sectional study reviewed and approved by the Facultad de Medicina Ethics Committee at the Universidad de Antioquia, Medellín, Colombia (Record 005; 31^st^ March 2016). The approved protocol included the activities performed in the current retrospective study. Before starting any procedure, all participants provided their written informed consent or informed assent (women <18 years) for the use of their samples for retrospective testing of malaria parasites. For participants <18 years old additional informed consent from parents or legal guardians was also obtained. The study was conducted in accordance with the Declaration of Helsinki and local rules and regulations of Colombia.

### Study area

**Fig 1:**
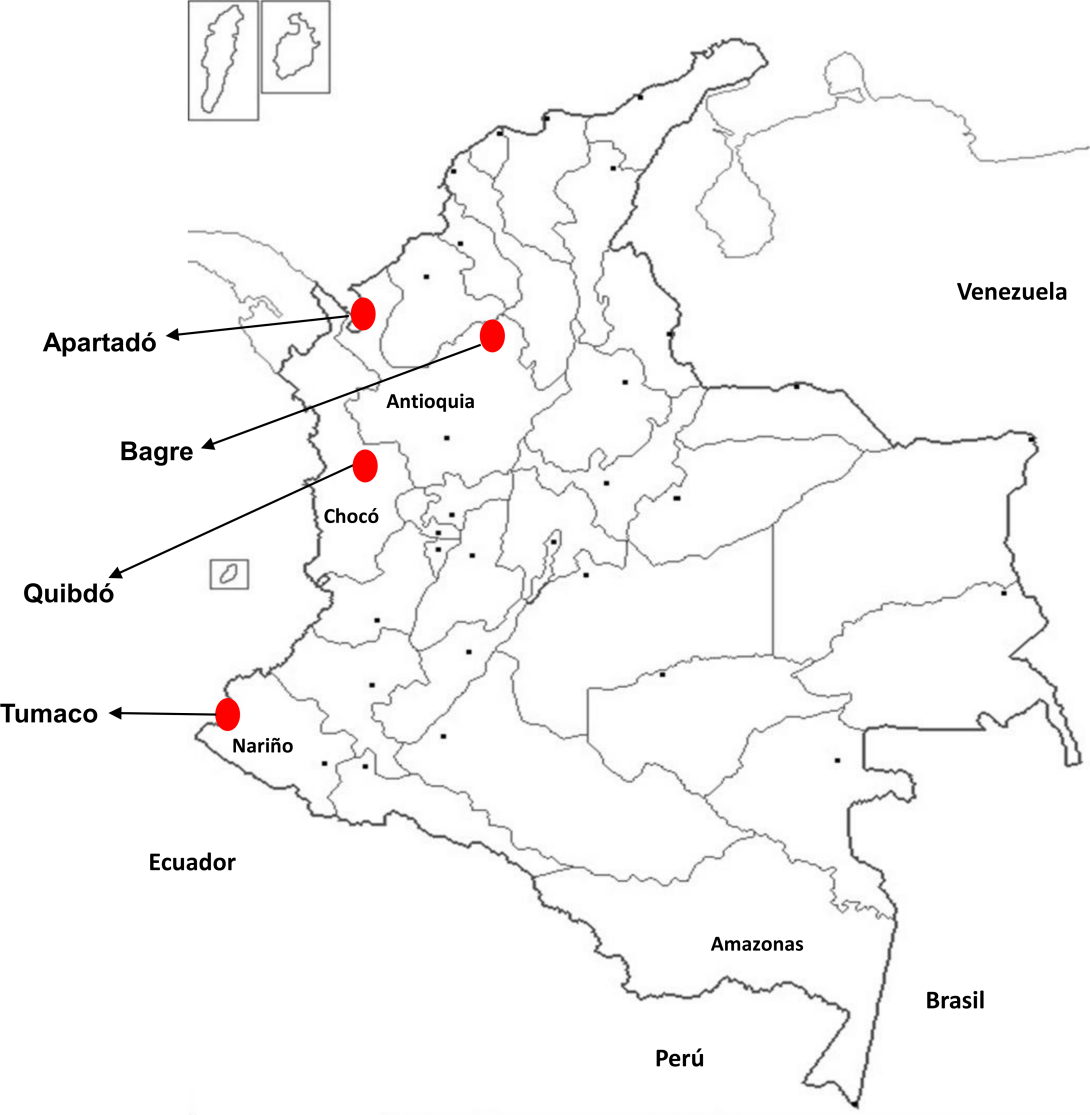
Study area. Map of Colombia showing the four study sites in red dots: Apartadó, Bagre, Quibdó and Tumaco.

The study was carried out in four Colombian malaria-endemic municipalities: El Bagre, Apartadó, Quibdó and Tumaco (Fig 1). El Bagre and Apartadó are located in Antioquia’s Department, in the northwest of Colombia, with *P*. *vivax* as the predominant *Plasmodium* specie (*spp*.) (70%). The municipality of Quibdó is located in the pacific region, in the Chocó’s Department, in the west of Colombia, with *P*. *falciparum* as the predominant parasite (80%). Tumaco is located in the pacific coast in Nariño´s Department, in the southwest of the country near the border with Ecuador, where the predominant specie is *P*. *falciparum* (90%).

Study sites were selected based on the incidence and number of symptomatic cases in general population reported during previous years in Colombia [28].

### Data and sample collection

Socio-demographic profiles, obstetric history and other clinical data were gathered at local hospitals using an interview-based questionnaire. Axillary temperature was also recorded using a digital thermometer. Peripheral blood (8 mL) was collected from each participant by venipuncture, using heparin tubes. When pregnant women were enrolled at delivery, placental blood (8 mL) was also collected, briefly, a small incision was made on the maternal side of the placenta, near the cord insertion area after a wash with saline (0.9%), placental blood accumulated at the place of insertion was collected by aspiration with a pasteur pipette.

### Sample processing

Blood samples were transferred to the local hospital’s laboratory. Thick and thin blood smears were prepared (80 μL of blood) and LM readings and Pf/Pv RDT testing (5 μL) were carried out on-site. Women with positive LM or Pf/Pv RDT results received free antimalarial treatment according to national treatment guidelines. A total of 100 μL of blood were stored at -80°C until transport to the reference laboratory at the Universidad de Antioquia in Medellín for HS-RDT and Pf RDT testing. In addition, 200 μL of blood were spotted on Whatman filter paper #3 (Fisher, Ref 1003–917), air-dried, and stored at room temperature in sealed bags with desiccant for nPCR testing in the reference laboratory.

### Diagnostic test procedures

#### Conventional rapid diagnostic test (Pf/Pv RDT)

The SD Bioline Malaria antigen Pf/Pv (Standard Diagnostics, Korea, 05FK80) was performed in local hospitals by trained staff, using fresh blood samples and according to manufacturer’s instructions. This test–referred through the document as Pf/Pv RDT**–**was able to distinguish between *P*. *falciparum*, *P*. *vivax* and mixed infections based on the detection of the following antigens: HRP2 and *P*. *vivax*-specific pLDH. The Pf/Pv RDT was selected because it is one of the most commonly used RDT in Colombia and is one of the top-10 performing HRP2 and pLDH tests for detecting *P*. *falciparum* and *P*. *vivax*, as reported by the World Health Organization (WHO)-FIND RDT evaluation program [17].

#### Highly sensitive rapid diagnostic test (HS-RDT)

Frozen blood samples were tested retrospectively in duplicate (two test performed by sample) by two independent trained researchers using the Alere Malaria Ag P.f RDT Ultra-Sensitive (Standard Diagnostics, Korea, 05FK140); referred here as HS-RDT. This test was performed according to manufacturer’s instructions. Test results were read after 20 minutes and the outcome was interpreted as invalid (no control line), positive (presence of the control and *P*. *falciparum* line), or negative (presence of the control line present, but no *P*. *falciparum* line). The final result (invalid, positive or negative) was based on the consensus of the two independent test. In case of discrepant results the test was repeated a third time to confirm results.

#### Conventional rapid diagnostic test for quality control (Pf RDT)

A second conventional RDT, in addition to the Pf/pv RDT, was used retrospectively in frozen blood samples, in order to discard antigen degradation due to sample freezing. The SD Bioline Malaria Ag P.f RDT (Standard Diagnostics, Korea, 05FK50), referred in this manuscript as Pf RDT, was used in duplicate by two independent trained researchers, according to manufacturer’s instructions. The Pf RDT is specific for HRP2 antigen and was selected on the basis of its performance in the WHO-FIND malaria RDT product testing programme [17]. The final result (invalid, positive or negative) was based on the consensus of the two independent readers. In case of discrepant results the test was repeated to confirm results.

#### Light microscopy (LM)

Field-stained thick and thin blood slides were read by an expert malaria microscopist according to national guidelines [29]. Parasitaemia was estimated against 200 leukocytes (assuming a standard value of 8,000 leukocytes/μL of blood) and was expressed as parasites/μL (p/μL). *P*. *falciparum* parasitaemia was calculated counting ring forms, while *P*. *vivax* parasitaemia was calculated counting all asexual forms. A sample was considered negative if after the examination of 200 microscopic fields at 100x magnification, no parasites were observed. As a quality control, a second reading was performed in all PCR positives samples and in 10% of negative ones. Discrepant results (positive vs. negative; parasitaemia difference > 50%; different species) were resolved by a third reading. The final parasitaemia was the average of all readings, calculated as previously described: parasite densities was calculated by averaging the two counts and in the blood smears with non-concordant results, parasite density was calculated by averaging the two most concordant counts [30].

#### Nested PCR (nPCR)

Deoxyribonucleic acid (DNA) was extracted from half blood-spot filter using QIAamp DNA Mini Kit (Qiagen, Germany, Ref 51306), according to manufacturer's instructions. Nested PCR was performed as a two-step procedure, following the protocol described by Singh et. al 1999 with minor modifications [31]. This protocol consists of a universal PCR followed by nested species-specific PCR to detect the 18S ribosomal gene of *P*. *falciparum*. Each 20 μL reaction mix contained 2 μL of the eluted DNA. Positive and negative reaction controls were included. Amplification products were resolved in a 1.5% agarose gel stained with GelRed ™ (Biotium, ref. 41003, United States) and visualized under ultraviolet light. The current nPCR method was selected as the reference test based on its availability to detect *Plasmodium* low-density infections (LoD = 1 p/μL).

### Data management and statistical analysis

All data were collected using standardised questionnaires and forms and entered into a Microsoft Access database and Excel sheet. Data analysis was carried out using SPSS version 23.0. Infection prevalence was derived and 95% confidence intervals (CI) were calculated when applicable. Sensitivity, specificity, positive predictive value (PPV), and negative predictive value (NPV) were determined for LM (comparator test), Pf/Pv RDT (comparator test), Pf RDT (comparator test), and HS-RDT (index test), using nPCR as the reference standard. Kappa coefficient was calculated to assess the agreement among different diagnostic methods, accounting for random effect. Complementary analysis for diagnostic test accuracy was carried out in two subgroups of participants (asymptomatic and symptomatic).

### Definitions

Low-density infection [32] was defined as *Plasmodium spp*. infection detected by nPCR but not by LM. Symptomatic infections were defined as infections detected by LM, HS-RDT, conventional RDTs, or nPCR in pregnant women with fever (axillary temperature ≥ 37.5°C) or history of fever in the last 3 days [12]. Asymptomatic infections were defined as infections detected by LM, HS-RDT, conventional RDTs, or nPCR in pregnant women without fever or history of fever in the last 3 days [12]. Symptomatic pregnant women were defined as study participants with fever (axillary temperature ≥ 37.5°C) or history of fever in the last 3 days, regardless if they were positive for malaria. Asymptomatic pregnant women were defined as study participants without fever or history of fever in the last 3 days, regardless they were positive for malaria.

## Results

### Study participants baseline characteristics

**Fig 2:**
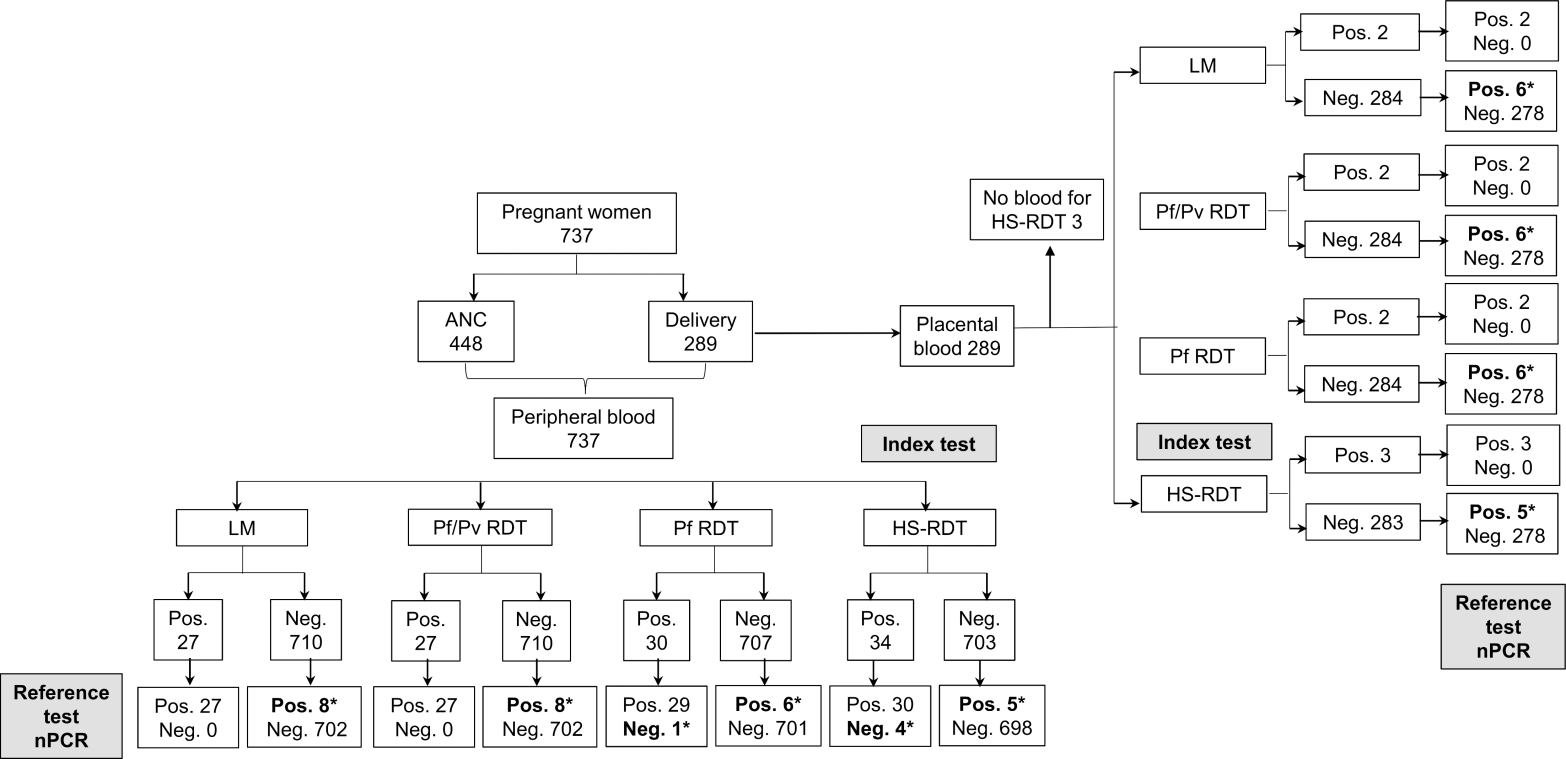
Study participant flow and testing results. The chart shows the total number of pregnant women recruited during antenatal care visits and at delivery, the number of peripheral and placental blood samples collected, as well as the overall number of malaria infections detected by each test. *Discrepant results when compared with the reference test ANC (antenatal care); Pos. (positive); Neg. (negative); LM (light microscopy); Pf (*P*. *falciparum*); Pv (*P*. *vivax*); RDT (rapid diagnostic test); HS-RDT (highly sensitive rapid diagnostic test); nPCR (nested polymerase chain reaction).

Overall, 737 pregnant women were recruited across all study sites (Fig 2), 60.8% (448/737) during ANC and 39.2% (289/737) at delivery. Socio-demographic characteristics, obstetric and clinical information, as well as history of malaria are shown in Table 1. The median age of participants was 23 years (range 15–45), being 41.0% (302/737) primigravida. At enrollment, 3.3% (24/737) of pregnant women presented fever (axillary temperature >37.5°C) and 7.1% (52/737) reported history of fever within the last three days. The percentage of participants reporting history of malaria within current pregnancy was 4.2% (31/737).

**Table 1:** Baseline characteristics of study participants at enrolment.

	Total (N = 737)	ANC (N = 448)	Delivery (N = 289)
Age (years): median (IQR)	23 (20–28)	23 (19–28)	24 (21–28)
Gestational age (weeks): median (IQR)	30 (17–38)	20 (13–27)	38 (37–39)
Gravidity: median (IQR)	1 (0–2)	1 (0–2)	1 (0–1)
Primigravida: N (%)	302 (41.0)	153 (34.2)	149 (51.6)
Fever at enrollment: N (%)	24 (3.3)	15 (3.3)	9 (3.1)
History of fever (72 hours): N (%)	52 (7.1)	30 (6.7)	22 (7.6)
Malaria within current pregnancy: N (%)	31 (4.2)	20 (4.5)	16 (5.5)

ANC (antenatal care); IQR (Interquartile range); N (sample size).

### Infection rate by diagnostic test

In peripheral blood samples (Table 2), the overall positivity rate detected by the HS-RDT (4.6%; 34/737) was similar to nPCR (4.7%; 35/737). Thirty infections were observed by Pf RDT (4.1%; 30/737) and twenty-seven by Pf/Pv RDT and LM (3.7%; 27/737). Parasite density values obtained by LM were 2,417 rings/μL [interquartile range 729–4,052 rings/μL of blood (range 140–41,211)]. Most infections (30/35 for nPCR) were observed in the ANC group.

**Table 2:** *P*. *falciparum* positivity rate by diagnostic test in peripheral blood samples collected during ANC visits and at delivery.

Peripheral blood; N (%)	Total (N = 737)	ANC (N = 448)	Delivery (N = 289)
LM	27 (3.7)	27 (6.0)	0 (0.0)
Pf/Pv RDT	27 (3.7)	27 (6.0)	0 (0.0)
Pf RDT	30 (4.1)	27 (6.0)	3 (1.0)
HS-RDT	34 (4.6)	29 (6.5)	5 (1.7)
nPCR	35 (4.7)	30 (6.7)	5 (1.7)
Low-density infections	8 (1.1)	3 (0.7)	5 (1.7)

ANC (antenatal care); LM (light microscopy); Pf (*P*. *falciparum*); Pv (*P*. *vivax*); RDT (rapid diagnostic test); HS-RDT (highly sensitive rapid diagnostic test); nPCR (nested polymerase chain reaction); N (sample size).

Regarding placental specimens (Fig 2), the overall infection rate, as determined by nPCR, was 2.8% (8/286). The percentage of *P*. *falciparum* positive cases detected by the HS-RDT was 1% (3/286). LM, Pf/Pv RDT and Pf RDT showed a positivity rate of 0.7% (2/286). The parasitaemia of the two placental positive samples detected by LM was 160 and 200 rings/μL of blood. Low-density infections were observed in both, peripheral (1.1% 8/737) and placental (2.1%; 6/286) blood.

### Performance of diagnostic tests in peripheral blood

Using nPCR as the reference standard (Table 3), the HS-RDT showed slightly better sensitivity [85.7% (95% CI = 70.6–93.7)], when compared with LM [77.1% (95% CI = 61.0–87.9)], conventional Pf/Pv RDT [77.1% (95% CI = 61.0–87.9)], and Pf RDT [82.8% (95% CI = 67.3–91.9)], but the differences were no significant. Both LM and Pf/Pv were highly specific (100.0%). Pf RDT and HS-RDT displayed few false positive results when compared with nPCR. The false positive rate for Pf RDT and HS-RDT, defined as the percentage of all negatives with a positive result, was 0.14% (95% CI = 0.0–0.8) and 0.57% (95% CI = 0.2–1.5), respectively.

**Table 3:** Accuracy of LM, Pf/Pv RDT, Pf RDT, and HS-RDT for the diagnosis of *P*. *falciparum* infections in peripheral blood of pregnant women.

		PCR			Value (95% CI)				
		(+)	(-)	Total	Sensitivity	Specificity	PPV	NPV	Kappa
**LM**	(+)	27	0	27	77.1%	100.0%	100.0%	98.9%	0.9
	(-)	8	702	710	(61.0–87.9)	(99.5–100.0)	(87.5–100.0)	(97.8–99.4)	(0.8–1.0)
**Pf/Pv RDT**	(+)	27	0	27	77.1%	100.0%	100.0%	98.9%	0.9
	(-)	8	702	710	(61.0–87.9)	(99.5–100.0)	(87.5–100.0)	(97.8–99.4)	(0.8–1.0)
**Pf RDT**	(+)	29	1	30	82.8%	99.9%	96.7%	99.1%	0.9
	(-)	6	701	707	(67.3–91.9)	(99.2–100.0)	(83.3–99.4)	(98.2–99.6)	(0.8–1.0)
**HS-RDT**	(+)	30	4	34	85.7%	99.4%	88.2%	99.3%	0.9
	(-)	5	698	703	(70.6–93.7)	(98.5–99.8)	(73.4–95.3)	(98.3–99.7)	(0.8–1.0)

LM (light microscopy); Pf (*P*. *falciparum*); Pv (*P*. *vivax*); RDT (rapid diagnostic test); HS-RDT (highly sensitive rapid diagnostic test); nPCR (nested polymerase chain reaction); (+) (positive); (-) (negative); PPV (positive predictive value); NPV (negative predictive value); CI (confidence interval)

Regarding test performance in symptomatic (61) and asymptomatic (649) pregnant women, all methods showed sensitivity greater than 95% and specificity of 100% when detecting symptomatic infections (21/61) (Table 4). There was one symptomatic case not detected by any of the three HRP2-based RDTs, but confirmed by LM (4,018 rings/μL) and nPCR. In asymptomatic participants, LM and Pf/Pv RDT missed half of the infections detected by nPCR (14/30). Although Pf RDT and HS-RDT also missed some cases, the HS-RDT showed higher sensitivity [71.4% (CI 95% = 45.4–88.3)] compared with LM and Pf/Pv RDT [50% (CI 95% = 27.8–73.2)], but differences were no significant. Overall, 27 participants with no data on temperature or fever history were excluded for this analysis.

**Table 4:** Performance of LM, Pf/Pv RDT, Pf RDT, and HS-RDT for detecting *P*. *falciparum* in peripheral blood of symptomatic and asymptomatic pregnant women.

					Value (95% CI)			
**Symptomatic (N = 61)**	**Test**		**PCR**		**Sensitivity**	**Specificity**	**PPV**	**NPV**
			(+)	(-)				
	**LM**	(+)	19	0	95.2% (77.3–99.2)	100.0% (91.2–100.0)	100.0% (83.9–100.0)	97.6% (87.4–99.6)
		(-)	0	42				
							
	**Pf/Pv RDT**	(+)	18	0	95.2% (77.3–99.2)	100.0% (91.2–100.0)	100.0% (83.9–100.0)	97.6% (87.4–99.6)
		(-)	1	42				
							
	**Pf RDT**	(+)	18	0	95.2% (77.3–99.2)	100.0% (91.2–100.0)	100.0% (83.9–100.0)	97.6% (87.4–99.6)
		(-)	1	42				
							
	**HS-RDT**	(+)	18	0	95.2% (77.3–99.2)	100% (91.2–100.0)	100.0% (83.9–100.0)	97.6% (87.4–99.6)
		(-)	1	42				
**Asymptomatic (N = 649)**	**LM**	(+)	7	0	50.0% (26.8–73.2)	100.0% (99.4–100.0)	100.0% (64.6–100.0)	98.8% (97.7–99.4)
		(-)	7	635				
							
	**Pf/Pv RDT**	(+)	7	0	50.0% (26.8–73.2)	100% (99.4–100.0)	100% (64.6–100.0)	98.8% (97.7–99.4)
		(-)	7	635				
							
	**Pf RDT**	(+)	9	0	64.3% (38.8–83.7)	100.0% (99.4–100.0)	100.0% (70.1–100.0)	99.2% (98.1–99.7)
		(-)	5	635				
							
	**HS-RDT**	(+)	10	3	71.4% (45.4–88.3)	99.5% (98.6–99.8)	76.9% (49.7–91.8)	99.4% (98.3–99.7)
		(-)	4	632				

LM (light microscopy); Pf (*P*. *falciparum*); Pv (*P*. *vivax*); RDT (rapid diagnostic test); HS-RDT (highly sensitive rapid diagnostic test); nPCR (nested polymerase chain reaction); (+) (positive); (-) (negative); PPV (positive predictive value); NPV (negative predictive value); CI (confidence interval); N (sample size).

### Performance of diagnostic tests in placental blood

Using nPCR in placental blood as the reference test, the sensitivity of HS-RDT was slightly higher [37.5% (95% CI = 13.7–69.4)], compared with LM and conventional RDTs [25.0% (7.1–59.1)]. The HS-RDT detected one additional infection (3/8) compared with LM and conventional RDTs (2/8). Using nPCR in placental blood as the reference standard, neither LM nor Pf/Pv RDT were able to detect any placental infection in peripheral blood, while nPCR, HS-RDT and Pf RDT detected 62.5% (95% CI = 30.6–86), 37.5% (95% CI = 13.7–69.4), and 25% (95% CI = 7.1–59.1) of placental infections respectively. However the number of infected placentas detected by the reference test was so low (n = 8), that it did not allow to draw conclusions from such a small number of positives.

## Discussion

To the extent of our knowledge, this is the first study evaluating the accuracy of the new HS-RDT for the detection of *P*. *falciparum* infections in samples from pregnant women in low-transmission settings. Using nPCR as the reference standard, the HS-RDT showed a slightly improved sensitivity for detecting malaria in peripheral and placental specimens, compared with LM and conventional RDTs. This improvement was particularly evident when screening asymptomatic participants and for identifying low-density parasite infections. In addition, the study demonstrates the need of further investigations of usefulness of the new test for detecting placental infections in peripheral blood using a large sample of placental infections.

Overall, 4.7% of the study participants were found to harbor malaria parasites in peripheral blood. This observation was in line with recent studies conducted in Colombia, showing a prevalence of *P*. *falciparum* mono-infections of 3.0–4.1% [6,7]. When using nPCR as the reference test, HS-RDT displayed a slightly improvement in sensitivity (85.7%), compared with LM (77.1%) and conventional RDTs (77.1–82.8%). Similar results have been reported in non-pregnant population, where HS-RDT sensitivity was higher than RDT in both, high- and low-transmission settings [24].

As previously reported, conventional RDTs performed similar to LM when detecting peripheral *P*. *falciparum* infections in pregnant women [15,19–22,33,34]. Accuracy discrepancies between the two conventional RDTs used in this study could be explained by the different panel detection scores observed in the WHO-FIND malaria RDT testing programme (95% Pf RDT vs. 92% Pf/Pv RDT for *P*. *falciparum*) [35]. Furthermore both, LM and conventional RDTs, missed a higher number of low-density infections than nPCR. This observation emphasized the underestimation of malaria infections by tests routinely used in pregnant women attending ANC visits and at delivery. In this sense, further studies are needed to guide recommendations on the use of the HS-RDT in pregnant women with inherently low-density *P*. *falciparum* levels.

The accuracy of the HS-RDT for screening *P*. *falciparum* infections in symptomatic and asymptomatic women was also evaluated in the current study. In agreement with previous findings, the performance of conventional RDTs and LM was superior in symptomatic than asymptomatic cases, showing high sensitivity (>95%) and specificity (100%), probably due to higher parasite densities in febrile infections [21,33]. Nevertheless, the HS-RDT showed an improved sensitivity in afebrile participants (71.4%), where LM and conventional RDTs missed 50% of infections. Although the clinical relevance of pregnant women as asymptomatic parasite reservoir is not yet understood, asymptomatic carriers could remain as reservoirs for malaria transmission [9,11]. In this sense, the HS-RDT could be a promising tool for managing MiP during ANC visits and at delivery, particularly in low-transmission settings where intermittent preventive treatment during pregnancy is not recommended and only positive malaria cases are treated [19].

It is worth noting that one of the symptomatic cases detected by LM in Apartadó was negative by all three RDTs regardless being a specimen with high parasite density. This result suggests the potential presence of *pfhrp2/pfhrp3* deletions, as previously reported in the south of Colombia and in the border of Peru [36,37]. Certainly, the current configuration of the HS-RDT does not allow identifying parasites with *pfhrp2* deletions, which might be one of the current limitations of the test.

Although sensitivity is a key feature to ensure end-users that the HS-RDT is unlikely to miss any malaria infection, the PPV is also an important parameter to take into account. Overall, the HS-RDT showed lower PPV compared with LM (88% vs. 100%), especially in afebrile women. This observation could be related to the presence of false positive results, probably due to HRP2 persistence in blood [19,24,38] or cross-reaction with other proteins such as rheumatoid factor [16,24,39]. Further research is needed to evaluate the HRP2 HS-RDT-based positivity time-course in MiP, as well as potential cross-reactivity limitations.

Regarding placental specimens, the overall infection rate confirmed by nPCR of 2.8% was lower than in peripheral blood samples and in line with latest prevalence reported in the area [6]. Given that access to placental tissue and blood during pregnancy is practically impossible, an ideal diagnostic test should be able to detect placental infections in peripheral blood. The current results showed that all test performed in peripheral blood underestimated placental infections detected by nPCR, probably due to parasite sequestration in placenta [40]. In the study areas, the routine tests used for screening malaria in peripheral blood (i.e. LM and Pf/Pv RDT) failed to detect all placental infections. Instead, the HS-RDT and Pf RDT improved the diagnosis of placental malaria, detecting 37.5% and 25% infections, respectively, however the low number of infected placentas make it difficult to draw any conclusion. In light with the above findings, further studies are needed to assess the usefulness of the new HS-RDT for detecting placental malaria.

### Study limitations

The low number of malaria-positive samples was the main limitation of this study. Sample size was estimated to meet the main objective of a larger cross-sectional project aiming to characterize asymptomatic infections in Colombian pregnant women and to assess their impact in malaria-related adverse pregnancy outcomes. Despite the sample size for the current retrospective sub-study was too small to provide statistically significant accuracy values, HS-RDT displayed an improved sensitivity compared with LM and RDTs for screening MiP. Future studies should consider a larger sample size.

While study staff errors cannot be excluded at local hospitals, training was provided before starting the study, making operator factors an unlikely cause of poor LM and Pf/Pv RDT performance. Moreover, the HS-RDT was performed in duplicate, in parallel to a quality control test (Pf RDT), and in a reference laboratory by highly trained staff. However, the fact that HS-RDT was run in laboratory conditions with expert readers does not necessary illustrates performance under field conditions. In this regard, further studies showing the usefulness of the HS-RDT detecting MiP in remote settings, outside reference centers are needed. Finally, the low prevalence of malaria cases in this study did not allow an evaluation of the impact of missed infections in pregnancy and birth outcomes.

## Conclusion

The improved sensitivity of the HS-RDT over LM and conventional RDTs for detecting gestational and placental malaria, particularly in asymptomatic women, indicates the potential value of this test for managing malaria in pregnancy. This performance should be confirmed by larger studies under field conditions, using finger-prick blood specimens. The cost-effectiveness of integrating this new tool in the maternal health programs in low-transmission settings should also be evaluated. Finally, the impact that this highly sensitive tool may have in malaria-related adverse pregnancy and birth outcomes should be investigated.
